# Bio‐Mimic, Fast‐Moving, and Flippable Soft Piezoelectric Robots

**DOI:** 10.1002/advs.202300673

**Published:** 2023-05-10

**Authors:** Erdong Chen, Yiduo Yang, Mengjiao Li, Binghang Li, Guijie Liu, Weilei Mu, Rong Yin

**Affiliations:** ^1^ College of Engineering Ocean University of China Qingdao 266100 China; ^2^ Textile Engineering, Chemistry and Science Wilson College of Textiles North Carolina State University Raleigh NC 27695 USA

**Keywords:** fast moving, piezoelectric, resonance, soft robots, spiral structure

## Abstract

Cheetahs achieve high‐speed movement and unique athletic gaits through the contraction and expansion of their limbs during the gallop. However, few soft robots can mimic their gaits and achieve the same speed of movement. Inspired by the motion gait of cheetahs, here the resonance of double spiral structure for amplified motion performance and environmental adaptability in a soft‐bodied hopping micro‐robot is exploited. The 0.058 g, 10 mm long tethered soft robot is capable of achieving a maximum motion speed of 42.8 body lengths per second (BL/s) and a maximum average turning speed of 482° s^−1^. In addition, this robot can maintain high speed movement even after flipping. The soft robot's ability to move over complex terrain, climb hills, and carry heavy loads as well as temperature sensors is demonstrated. This research opens a new structural design for soft robots: a double spiral configuration that efficiently translates the deformation of soft actuators into swift motion of the robot with high environmental adaptability.

## Graphical abstract

This paper presents a new soft robot design with a double spiral that moves at a speed of 42.8 body lengths per second and maintains movement even when flipped over. The robot is small, weighs 0.058 g, and can navigate complex terrain, climb hills, carry heavy loads, and temperature sensors. This new design offers efficient and adaptable movement for soft robots.

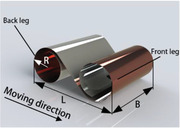

## 1 Introduction

Traditional rigid robots are composed of rigid materials and components such as metals. They are driven by motors and hydraulics, with low work‐to‐weight ratio, high inertia, and limited degrees of freedom. Soft robots are composed of soft materials with low stiffness and are driven by soft actuators. Several different types of soft actuators have been developed such as light actuators,^[^ ^1^, ^2^, ^3^, ^4^, ^5^, ^6^, ^7^ ^]^ electrical actuators,^[^ ^8^, ^9^, ^10^, ^11^, ^12^, ^13^, ^14^, ^15^, ^16^, ^17^, ^18^, ^19^, ^20^, ^21^, ^22^, ^23^, ^24^, ^25^, ^26^ ^]^ thermal actuators,^[^ ^27^, ^28^, ^29^, ^30^, ^31^, ^32^, ^33^, ^34^, ^35^, ^36^ ^]^ magnetic actuators,^[^ ^37^, ^38^, ^39^, ^40^, ^41^, ^42^, ^43^, ^44^, ^45^, ^46^, ^47^ ^]^ and fluidic actuators.^[^ ^48^, ^49^, ^50^, ^51^, ^52^, ^53^ ^]^ Soft actuators and body structures enable more degrees of freedom. They can be integrated and fused, similar to biological systems. Soft‐bodied robots inspired by natural mollusks have shown great advantages. Bird‐like or fish‐like flapping soft robots driven by fluid^[^ ^48^ ^]^ and electric^[^ ^8^, ^9^, ^10^, ^11^, ^12^, ^13^ ^]^ have shown high propulsion efficiency. Worm‐like crawling soft robots have been developed for underground exploration,^[^ ^54^ ^]^ pipeline inspection,^[^ ^10^ ^]^ and gastrointestinal endoscopy,^[^ ^37^, ^38^ ^]^ showing good environmental adaptability. Soft‐bodied robots that can change locomotion mode from crawling to rolling have the advantages of high efficiency and mobility.^[^ ^35^, ^36^, ^37^, ^38^, ^39^, ^40^, ^41^, ^42^, ^43^, ^44^, ^45^, ^46^, ^47^, ^48^, ^49^, ^50^, ^51^, ^52^ ^]^ However, most of the currently developed soft robots have motion speeds < 10 BL s^−1^, in which a huge gap exists compared with biological counterparts. Therefore, great challenges remain in developing soft robots with high motion performance and good environmental adaptability.

To address the challenge, we developed a soft‐bodied hopping micro‐robot exploiting the resonance of double spiral structure for amplified motion performance and environmental adaptability. Vibration is a fast motion that is often observed in nature (e.g., mosquitoes^[^ ^55^ ^]^) and in our daily lives (e.g., strings). Resonance has been utilized to amplify the displacement output to improve energy harvesting performance.^[^ ^56^, ^57^ ^]^ Moreover, resonance can also be used as a means of power transmission to convert oscillating energy into a desired periodic motion.^[^ ^58^ ^]^

Recently, harnessing vibration for high‐performance soft robots has attracted growing interest in addressing soft body compliance‐related issues such as slow response and small output forces. This is particularly attractive for jumping and hopping soft robots, as jumping and hopping tend to require a higher frequency of motion and higher friction than crawling and rolling motions. Furthermore, the vibration‐driven locomotion mechanism allows for flexible structural design and facilitates the realization of compact structures without the need for a transmission mechanism. As a result, vibration‐based robots are also easier to miniaturize to explore narrow spaces. Vibration‐based micro‐robots can be realized by lead zirconate titanate (PZT) actuators^[^ ^59^, ^60^ ^]^ and dielectric elastomer actuators,^[^ ^25^, ^26^, ^27^, ^28^, ^29^, ^30^, ^31^, ^32^, ^33^, ^34^, ^35^, ^36^, ^37^, ^38^, ^39^, ^40^, ^41^, ^42^, ^43^, ^44^, ^45^, ^46^, ^47^, ^48^, ^49^, ^50^, ^51^, ^52^, ^53^, ^54^, ^55^, ^56^, ^57^, ^58^, ^59^, ^60^, ^61^ ^]^ which often exhibit faster motion. However, these two actuators usually require complex excitation sources to generate specific signals with high voltage or high frequency. For example, PZT‐driven vibrating robots usually have very small amplitudes in the µm range and require frequencies above 10 kHz to reach resonance. DEA‐driven vibrating robots do not require excessively high excitation frequency, but the excitation voltage often need to reach several thousand volts or more. In addition, robots based on vibration mechanisms usually have a backward motion phase within a cycle, limiting further improvements in efficiency and speed.^[^ ^62^ ^]^

Polyvinylidene fluoride (PVDF) as a smart piezoelectric material stands out among various soft actuators with its low power consumption, high efficiency, and fast response. Compared to the traditional PZT actuators with drawbacks such as toxicity, high excitation frequency, small amplitude, and brittleness, PVDF drivers have the advantages of softness, low excitation frequency, large strain, nontoxicity, etc. Jumping and hopping robots based on PVDF actuators can generate vibrations of several hundred Hz simply by bending their body parts without additional energy storage processes, which can boost the frequency of jumps and thus achieve movement speeds of up to 20 BL/s^−1[18]^ and turning speeds of 28 BL s^−2^.^[^ ^19^ ^]^ However, 1)They will have a significant proportion of obstructive gait which is the opposite to the direction of movement when the frictional force is generated by contact with the ground during high‐speed movement, which limits the further improvement of its movement speed. 2) Most of the previously mentioned soft robots lack the ability to walk continuously after flipping due to sudden changes in the environment (e.g., external forces or complex terrain). In fact, changes in working conditions often occur in practice, leading to robot motion failure, which limits the application of jumping and hopping soft robots. This problem, to our knowledge, lacks investigation, and corresponding soft robots overcoming this issue have not been developed. In general, optimizing the kinematic gait to further increase speed and preventing flipping to expand its application scenarios are two major engineering challenges for soft robots that implement jumping and hopping locomotion modes based on vibration.

In nature, cheetahs are footed mammals that move by hopping and jumping. They achieve a unique locomotor gait by contracting and expanding their limbs during gallop, with a maximum locomotor speed of 29 m s^−1^ (25 BL s^−1^).^[^ ^63^, ^64^, ^65^ ^]^ Inspired by cheetah motion gait, we report a bio‐mimic, fast‐moving, and flippable soft piezoelectric robot (BFFSPR) that efficiently convert bending of a flexible piezoelectric actuator into motion using the resonance of the double spiral shape structure. The BFFSPR demonstrates excellent performance by matching the gait that occurs during hopping and jumping with the friction force obtained to facilitate movement. The BFFSPR achieves a maximum average speed of 42.8 BL s^−1^ at its first resonant frequency, which is more than twice the speed of the fastest published piezoelectric soft robot. By connecting two robots in parallel, it also exhibits superior turning performance to that of existing soft robots with a maximum average turning speed of 482° s^−1^. We also demonstrated applications such as transporting a load 8 times of its own gravity, climbing hills, and carrying temperature sensors to detect high‐temperature environments. In addition, different from conventional rigid‐footed robots that rely on sensors and control system calculations to prevent flipping, our robot relies on the special vibration pattern of the centrosymmetric double spiral, which allows it to move at a high speed of 16 BL s^−1^ even after flipping, without additional complex control method. Finally, we showed the potential of BFFSPR to face a complex environment such as uneven terrains.

## 2 Result and Discussion

### 2.1 Structure and Working Principle of BFFSPR

**Figure 1:**
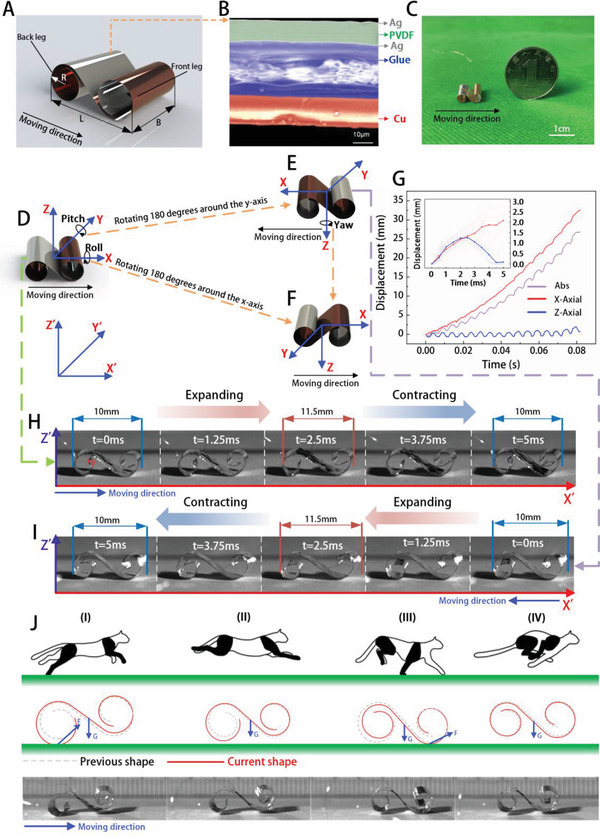
The prototype and working mechanism. A) 3D schematic of the BFFSPR. B) Scanning electron microscopy (SEM) image showing the cross‐sectional view of BFFSPR. C) Optical photos of the robot's appearance. D) Schematic diagram of the BFFSPR before flipping, where the local coordinate system *X*‐axis is the direction of robot motion. E) A diagram of the BFFSPR after it has been flipped around the pitch angle, where the *X*‐axis direction is opposite to that before the flipping. F) Schematic diagram of BFFSPR after flipping around the roll angle, where the X‐axis direction is the same as before flipping. G) Tracking the displacement change of point p continuously over 16 motion cycles, the inset is the change in displacement of point p in each cycle. H) Photograph of the BFFSPR before flipping in one drive cycle, with the direction of motion in the positive direction of the *X*′ axis. I) Photograph of the BFFSPR in one drive cycle after flipping at a pitch angle, with the direction of motion in the negative direction of the *X*′ axis. J) Comparing the characteristic gait of cheetahs and BFFSPR during locomotion.

**Figure** **1**A presents a 3D schematic illustration of the BFFSPR described in this study. The robot is composed of two symmetric, three‐quarter spirals with a radius of 2 mm, which form the front and back legs. The robot has a body length of 10 mm and a width of 10 mm. The BFFSPR is constructed using a piezoelectric actuator, which is shown in cross‐section in Figure 1B. The active layer of the actuator is a PVDF film with an Ag electrode attached using magnetron sputtering, while the passive layer is a Cu film. The two layers are connected together by glue, resulting in a total actuator thickness of 62 µm.

Exciting the PVDF film with voltage produces plane expansion through the inverse piezoelectric effect, while the passive layer limits expansion to induce bending deformation of the actuator. To achieve larger bending deformation, we explored the selection of PVDF film thickness using a combination of theoretical calculations and finite element analysis (FEA). Further details can be found in Text S1 and Figure S1, Supporting Information. Fabrication of the robot is simple and can be found in Text S5 and Figure S5, Supporting Information. The actual size of the BFFSPR is shown in Figure 1C in comparison with a one RMB coin.

Figure 1D illustrates the preflipping state of the BFFSPR, with its local *x*‐axis direction defining the forward motion direction of the robot and the positive direction of the global coordinate system *X*′. The BFFSPR can still move after flipping, although the direction of motion depends on the flipping axis. Figure 1E shows the BFFSPR after flipping around the Pitch angle, with the motion direction of the robot being the negative direction of the *X*' axis, opposite to that before flipping. Figure 1F shows the BFFSPR after flipping around the Roll angle, with the motion direction of the robot remaining the same as before flipping. These two flipped states can be transformed into each other by rotating around the Yaw angle.

We employed a high‐speed camera (FASTCAM Mini AX 100, Photon) operating at a sampling frequency of 4000 frames per second to capture the locomotion postures and positions of the prototype robot (under 200 V peak‐peak square voltage at 200 Hz, Video S1 and Video S2, Supporting Information). The locomotion behavior of the robot, both before and after flipping around the Pitch angle within one cycle of the applied excitation voltage, was analyzed based on continuous optical images, as shown in Figure 1H,I. During the first half cycle (0–2.5 ms), the robot is observed to be gradually expanding, while during the second half cycle (2.5–5 ms), the robot is gradually contracting (Video S1 and Video S2, Supporting Information). The red and blue lines of Figure 1G represent the horizontal and vertical displacements, respectively, of the tracking point p over 16 consecutive motion cycles. The robot accelerates from rest to nearly uniform motion in the first few cycles, with the front and back feet making contact with the ground and generating forces that affect motion during expansion and contraction. The inset in Figure 1G shows the average *X* and *Z*‐axis displacements of 16 cycles, which can be seen in Video S3, Supporting Information. It is worth noting that the deformation of the back foot part is significantly larger than that of the front foot, which contributes to the difference in motion performance of the robot before and after flipping. Cheetahs, along with many other mammals, utilize rotary gallop particularly at high speeds, which involves two phases of leap in the air (gathered flight and extended flight) and two phases of touchdown (forelimb body touchdown and hindlimb touchdown) per stride, as shown in Figure 1J.^[^ ^61^ ^]^ The gaits (I–IV) constitute a movement cycle of cheetah gallop. The length of the cheetah's body and the distance between the front and hind limbs change significantly during different locomotor gaits. We found that the BFFSPR also has very similar gaits during the high‐speed motion phase, as can be seen from the motion sketch in Figure 1J and the photographs captured by the high‐speed camera.

### 2.2 Locomotion Analysis of BFFSPR

**Figure 2:**
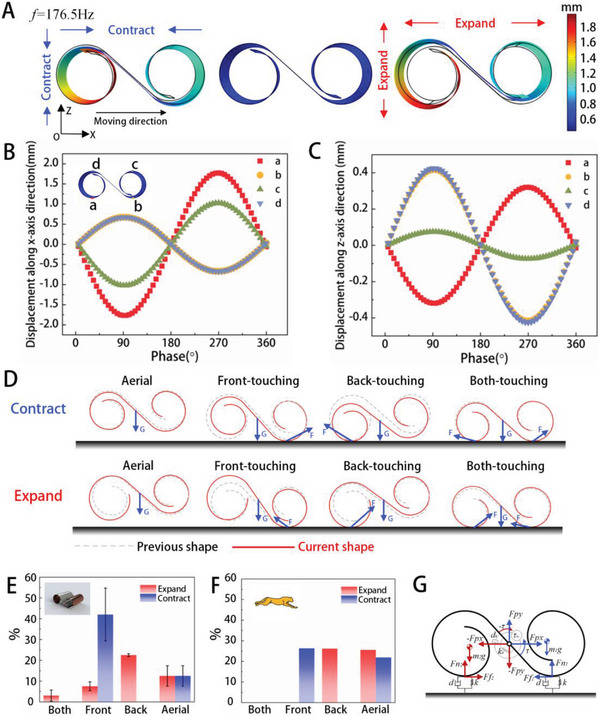
Motion mechanism analysis. A) FEM results of vibration mode analysis at first intrinsic frequency, showing the shape and deformation from the most contracted state to the most expanded state. B) FEM results of the displacement in the X‐axis of the four points (a, b, c, and d) that make contact with the ground during motion for one cycle. C) FEM results of the displacements in the *Z*‐axis for one cycle at the four points (a, b, c, and d) that make contact with the ground during the motion. D) Gait and force analysis with cross‐sectional views of corresponding contraction (top) and expansion (bottom) of the BFFSPR in eight different states (contraction in the air, expansion in the air, contraction at front foot touchdown, expansion at front foot touchdown, contraction at back foot touchdown, expansion at backfoot touchdown, contraction at both foot touchdown and expansion at both foot touchdown). E) The occurrence probability of each gait during the high‐speed motion of the robot. F) The occurrence probability of each gait of cheetahs. G) Simplified dynamic model based on two rigid bodies joined by a pin joint (both‐touching posture as an example) with a torsional spring‐damper system.

To explain the moving mechanism of the BFFSPR, the finite element method (FEM, COMSOL Multiphysics 5.6 Software) is utilized to obtain the resonant frequencies and vibration modes of the robot. The driving voltage applied to the PVDF film is ≈200 Vpp and mechanical boundary conditions are set to be free. The detailed simulation procedure and parameters are discussed in Text S2 and Tables S1 and S2, Supporting Information. The calculated resonant frequency of this bending mode is 176.5 Hz, and the maximum total displacement is ≈1.8 mm occurring at the end of the back leg. The robot contracts or expands in both length and height directions and the deformation of the back leg is obviously greater than that of the front leg, which is consistent with the posture captured by the high‐speed camera in Figure 1G. The changes of displacement along the *X*‐axis and *Z*‐axis of four top vertices (points a, b, c, and d) in one cycle are plotted in **Figure** **2**B,C. The calculated results indicate that front and back legs have different moving trajectories. The amplitude of point A is significantly larger than the amplitude of point B. Therefore, it can be inferred that the force acting between point A and the ground is also much larger than the force acting between point B and the ground.

Based on both FEM simulations and experimental observations, a total of eight distinct motion gaits were identified for the BFFSPR, as illustrated in Figure 2D. Among these, the back‐touching gait in expansion is the most effective for the robot's motion, and it also produces a torque effect on the robot to make the robot tilt in a certain way. Additionally, the Front‐touching gait in contraction was found to be effective for the robot's motion. We conducted an analysis of the friction between these two gait types and the ground, and further details can be found in Text S6 and Figure S7, Supporting Information. The probability of each gait occurring during the 70 motion cycles of the robot at high speed was calculated by the high‐speed camera and shown in Figure 2E. Among the eight possible gait patterns, front‐touching contraction, back‐touching expansion, aerial contraction, and aerial expansion account for a relatively large proportion, which is consistent with the probabilities of gait occurrence for cheetah gallop^[^ ^65^ ^]^ (Figure 2F). The robot's ability to achieve high‐speed motion can be attributed to two primary factors. First, the robot can achieve high speed by utilizing larger amplitude and higher frequency in its movements. Second, most of the gait patterns utilized by the robot are conducive to efficient motion. By employing a high‐speed camera, we were able to observe that the first vibrational mode is readily excited and plays a dominant role in shaping the robot's movements, while higher‐order modes can be disregarded.

To understand the motion characteristics of BFFSRP, we developed a dynamic mass‐spring model consisting of two rigid bodies (m_1_, m_2_) joined by a pin joint, as shown in Figure 2E (both‐touching posture as an example). A torsional spring–damper (k–d) at the pin joint is excited by a sinusoidally varying torque source (*m*) to represent the mechanical motions of the PVDF layer under the AC excitation. We modeled the ground contact at the front and back as a vertical spring‐damper (k–d) with a normal force in the vertical direction (*F*_n_) and a friction force in the lateral direction (*F*_f_). The details of the model are discussed in Text S4 and Figure S4, Supporting Information.

### 2.3 Performance Characterization

**Figure 3:**
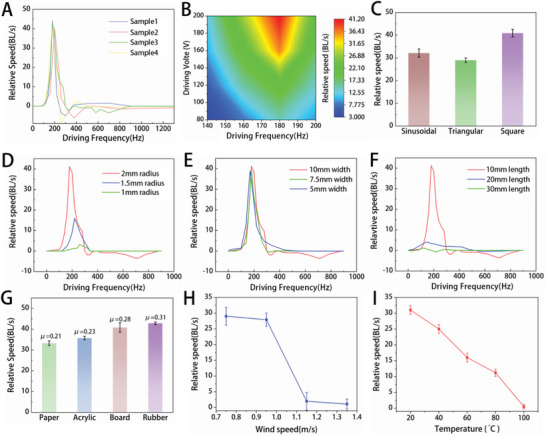
Motion performance characterization. A) Relationships between speed and frequency of several different robots moving on the board under 200 Vpp voltage excitation. B) The relationship between motion speed, excitation voltage amplitude, and excitation frequency. C) Maximum running speed of BFFSPR driven by different waveforms. D) Relationship between different spiral radius (*R*) and speed for a BFFSPR with a length and width of 10 mm. E) Relationship between different body widths (*B*) and speed for a BFFSPR with a length of 10 mm and a spiral radius of 2 mm. F) Relationship between different body lengths (*L*) and speed for a BFFSPR with a width of 10 mm and a spiral radius of 2 mm. G) Maximum running speed of BFFSPR on different substrates, including paper, acrylic plate, board, and rubber. H) Motion performance of the BFFSPR under different wind speeds. I) BFFSPR's motion performance at different heating temperatures, with paper as the friction interface. All error bars represent the standard deviation of three measurements.

In principle, a large‐amplitude oscillation driven at the resonant frequency should lead to significant deformation and greater forces, inducing faster speeds in a robotic system. To investigate this, we excited four prototype robots with square waves of 200 Vpp and varying frequencies, using a common board as the friction interface. The relationship between excitation frequency and speed was then determined, as illustrated in **Figure** **3**A. Our results confirmed that the speed of the robot at the first‐order resonant frequency was significantly higher than that at other excitation frequencies, supporting our initial hypothesis. Despite slight manufacturing discrepancies that resulted in slightly different resonant frequencies for each of the four robots (all within the range of 180 Hz–200 Hz), speeds above 40 BL s^−1^ were achieved. However, at higher frequencies, we observed negative velocity or reverse motion, which was due to a change in vibration mode to higher orders. This phenomenon is further explained in Text S3 and Figure S3, Supporting Information. Nevertheless, the vibration amplitude of higher‐order modes was found to be relatively small, resulting in poor motion performance, and was therefore not utilized as the primary working frequency and mode of the robot. The voltage amplitude is a crucial factor that affects the amplitude and motion speed of the robot. To investigate this relationship, we excited the robot using different square waves with voltages ranging from 80 V to 200 Vpp, and frequencies from 140 Hz to 200 Hz on the friction surface of the board. Our experimental results, as shown in Figure 3B, indicate that when the voltage is less than 80 V, it is challenging to achieve significant motion with a small vibration amplitude. As we were mindful of the durability of the PVDF film, we limited the voltage to below 200 Vpp to prevent electrical breakdown. We also studied the impact of different waveforms on robot speed at its 1st‐order resonant frequency under a voltage of 200 Vpp. As illustrated in Figure 3C, different waveforms resulted in different speeds, which may be related to the energy stored in the waveforms. The triangle waveform stored the least energy, and as such, the average speed of the robot movement was the smallest (29 BL s^−1^). The energy of the sine waveform was slightly higher than that of the triangle waveform, and the average speed of the robot was also slightly higher (32.1 BL s^−1^). Finally, the square waveform (50%) stored the most energy, resulting in the highest average speed of the robot (40.91 BL s^−1^).

We investigated the relationship between changes in the shape of the BFFSPR structure and its motion performance. Specifically, we studied the relationship between different spiral radius (*R*) and motion speed for a BFFSPR with a length and width of 10 mm, and the results are shown in Figure 3D. We found that when *R* decreased to 1.5 mm and 1 mm, the motion speed significantly decreased. The primary mechanism for BFFSPR's motion involves contraction and expansion deformation of its legs. Thus, when the size of the legs decreases, the corresponding deformation decreases, resulting in a decrease in motion speed. In addition, we examined the relationship between the width (B) of BFFSPR and its motion speed, while keeping the length and spiral radius constant at 10 mm and 2 mm respectively, as shown in Figure 3E. Our experimental results revealed that changes in the body width of BFFSPR do not have a significant effect on its speed. This finding can be attributed to the lack of significant changes in the deformation and resonance frequency of BFFSPR's legs, which are the primary mechanism for its motion. Furthermore, we studied the relationship between different body lengths (*L*) and motion velocity for a BFFSPR with a width of 10 mm and a spiral radius of 2 mm, as shown in Figure 3F. Our investigation revealed that an increase in body length (*L*) of BFFSPR results in a significant reduction in movement speed. This decrease in speed can be partly attributed to the decreased leg deformation relative to body size that occurs with increasing body length. Additionally, a longer body length can result in a decrease in the relative motion speed, calculated as the ratio of body length to movement speed.

Different contact interfaces will provide different friction forces to the robot. The coefficient of friction was experimentally measured by dragging the robot on different substrates, including paper (0.21), acrylic plate (0.23), board (0.28), and rubber (0.31). Figure 3G shows the maximum average motion speeds of the robot on paper (33.2 BL s^−1^), acrylic plate (35.7 BL s^−1^), board (40.8 BL s^−1^), and rubber (42.8 BL s^−1^) (Video S4, Supporting Information). As expected, the relative speed of the robot was found to be the fastest on rubber and slowest on paper. A higher coefficient of friction resulted in a faster relative speed of the robot. We also studied the motion performance of the BFFSPR under different wind speeds, and the experimental results showed that wind has an impact on the motion speed of the BFFSPR. As shown in Figure 3H, due to the small weight of the BFFSPR, it could no longer move forward and was blown away when the wind speed exceeded 1.35 m s^−1^. Experimental details are described in Text S7, Supporting Information. Wind did not cause any plastic deformation in the BFFSPR, so when the wind stopped acting on it, the BFFSPR was able to recover its previous motion performance. Temperature also had an impact on the motion performance of the BFFSPR. We placed the BFFSPR on a heating platform with an A4 paper and observed its motion performance at different heating temperatures, as shown in Figure 3I. When the temperature exceeded 100 °C, the BFFSPR became ineffective due to the increase in temperature.

### 2.4 After Flipping Locomotion Analysis

**Figure 4:**
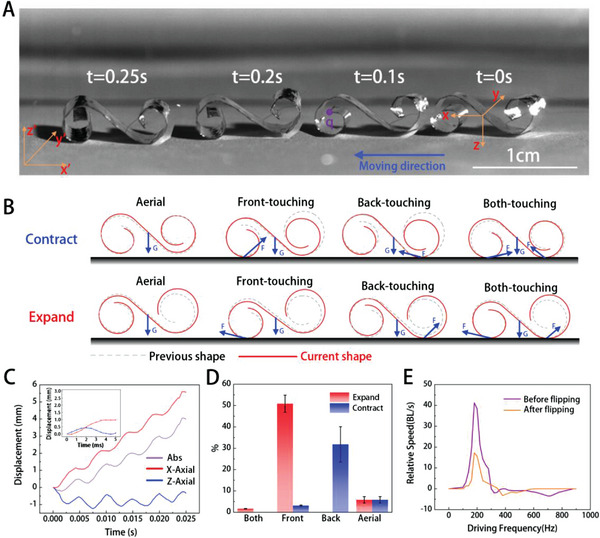
After flipping motion mechanism analysis. A) Real motion process of BFFSPR after flipping 180° around the Pitch angle according to frame‐by‐frame high‐speed video for 0.16 s with 200 V and first resonance frequency voltage. B) Gait and force analysis with cross‐sectional views of the BFFSPR after flipping 180° around the Pitch angle and corresponding contraction (top) and expansion (bottom) in eight different states (contraction in the air, expansion in the air, contraction at front foot touchdown, expansion at front foot touchdown, contraction at back foot touchdown, expansion at back foot touchdown, contraction at both foot touchdown and expansion at both foot touchdown). C) Changes in displacement of point q over 5 consecutive periods of motion. The inset is the average displacement changes of point q in each cycle. D) The occurrence probability of each gait of the robot after flipping under the excitation of the first resonance frequency and 200 Vpp. E) Comparison of the motion speeds of BFFSPR before and after flipping.

The BFFSPR's ability to move even after flipping allows it to face more complex environmental challenges. To study the motion mechanism of the BFFSPR after flipping, we flipped it 180° around the Pitch angle from the state shown in Figure 1C to the state shown in Figure 1E, and recorded its corresponding motion using a high‐speed camera, as shown in **Figure** **4**A. We observed that the moving direction of the robot was opposite to the direction before flipping, but the trend of speed increase from a slow start was consistent with that before flipping. The vibration mode of the BFFSPR after flipping was the same as that before flipping, as shown in Figure 2A, except that the points of contact with the ground changed from a and b to c and d. Using the same analysis method as before the robot flipped, we drew eight possible gait patterns and corresponding force analysis diagrams, which are shown in Figure 4B. We observed that the back‐touching gait facilitated the robot's motion during the contraction step, while the front‐touching gait facilitated the robot's motion during the expansion step.

We analyzed the probability of each gait in the 84 motion cycles of the robot in this motion mode, based on images taken by a high‐speed camera, as shown in Figure 4D. Two gaits were more probable: one where the front foot touched the ground during expansion and the other where the back foot touched the ground during contraction. The red and blue lines in Figure 4C indicate the horizontal and vertical displacements of the tracking point q on the robot's front leg for 5 consecutive motion cycles. The inset shows the average motion for 5 cycles, as demonstrated in Video S5, Supporting Information. In Video S2, Supporting Information, we observed that the amplitude of the front leg was significantly reduced compared to the back leg, which is consistent with the simulation results presented in Figure 2A. Figure 4E illustrates the relationship between the maximum speed and frequency of the robot excited by a voltage of 200 Vpp amplitude, before and after flipping. The maximum velocity of the robot, both before and after flipping, occurs at the first resonant frequency. However, the maximum velocity of the robot after flipping is 16 BL s^−1^, which is lower than the velocity before flipping. The reason is that the vibration amplitude of the contact points with the ground in the post‐flipping state (b,c) is significantly reduced compared to the contact points in the preflipping state (a,b), resulting in a smaller reaction force with the ground and slower motion. This is supported by both experimental observations (Figure 1C, Figure 4C) and simulation calculations (Figure 2A). Even though the motion speed after flipping is reduced compared to the preflipping state, it is still dominantly fast among existing soft robots.

### 2.5 Turning Performance of BFFSPR

**Figure 5:**
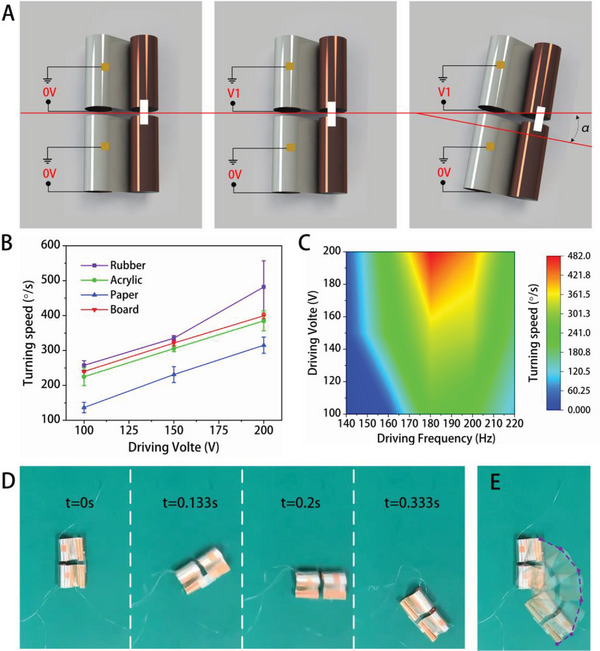
Turning results of the dual‐body BFFSPR. A) Schematic diagrams of the dual‐body BFFSPR turning process, which consists of the rest state, the starting state, and the turning state. In each voltage cycle, the robot turns by an angle of *α*. B) The relationship between turning speed and driving voltage on the four substrates. C) The relationship between turning speed and applied voltage and driving frequency. D) Still images illustrate the spot‐turning process of the robot on the board. E) Composite image of the trajectory for a continuous turning procedure on the board based on D).

To evaluate the agility of the robot, turning ability is also an important factor. We connected two BFFSPRs side by side using transparent tape to extend the bipedal soft robot into a quadrupedal soft robot, which can achieve turning motion (refer to Text S5 and Figure S6, Supporting Information, for details of the fabrication process). By applying an electrical excitation to one of the units, it vibrates and generates a forward friction force with the ground, while the other unit that is not excited generates the opposite friction force with the ground. The direction of the two friction forces is not in the same line, resulting in a steering behavior with a steering angle of *α* for each cycle, as shown in **Figure** **5**A,B shows the turning speed of the robot on four different friction interfaces, with paper resulting in the lowest average turning speed of 315° s^−1^, acrylic resulting in an average turning speed of 385° s^−1^, board resulting in an average turning speed of 399° s^−1^, and rubber resulting in the highest turning speed of 482° s^−1^ on average, which can be seen in Video S6, Supporting Information. The relationship between the turning speed and the driving voltage and driving frequency for the case of rubber as the interface is shown in Figure 5C. Similar to linear motion, the maximum turning speed also occurs at the voltage amplitude of 200 Vpp and the first resonance frequency of the robot. Figure 5D shows the real turning process according to frame‐by‐frame video on the board with 200 Vpp and the first resonance frequency, where the robot rotates 135° in 0.333 s. Figure 5E shows the trajectory of the robot during the turning process.

### 2.6 Multifunctionality: more than speed

**Figure 6:**
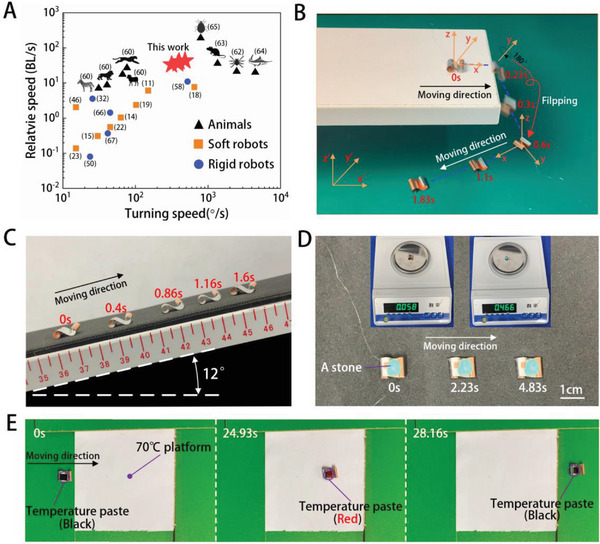
Fast, agile and versatile BFFSPR: flipping, climbing, loading, and detecting. A) Maximum running and turning speeds of representative animals, soft robots, and rigid robots. B) An image showing the ability of BFFSPR to move after falling and flipping from a high platform. C) BFFSPR climbing a 12° slope at 5.4 BL s^−1^. D) BFFSPR (0.058 g) carrying an oval stone (0.466 g) which is eight times of its own body weight, showing its load‐carrying capability. E) Temperature detection demonstration. The color of the temperature paste (55 °C) sticked to the robot changed from black to red when heated on the 70 °C platform.

Speed and agility are critical for survival strategies in many animal species. In **Figure** **6**A, we compare the relative speeds and turning speeds of our robots (represented by red stars) to those of animals, previously reported soft robots, and rigid robots.^[^ ^23^, ^24^ ^–^ ^34^, ^35^, ^36^, ^37^, ^38^, ^39^, ^40^, ^41^, ^42^, ^43^, ^44^, ^45^, ^46^, ^47^, ^48^, ^49^ ^–^ ^62^, ^63^, ^64^ ^–^ ^66^, ^67^, ^68^, ^69^, ^70^, ^71^ ^]^ The fastest relative speed of our robots reached 42.8 BL s^−1^ (absolute speed of 428 mm s^−1^ under square wave driving voltage of 200 Vpp at 180 Hz) which is more than twice as fast as the current insect‐scale piezoelectric soft robot (20 BL s^−1^).^[^ ^18^ ^]^ The combined mobility and agility performance of our robot is significantly superior to that of all other published robots, as indicated in Table S3, Supporting Information.

The unique double spiral structure and movement pattern of the BFFSPR allow it to maintain its ability to move even after flipping over, enabling it to tackle more complex environmental challenges. For instance, in Figure 6B, the robot can be seen moving along a high table under voltage excitation of 120Vpp and 180 Hz. It falls off the end of the table and flips with a 180° rotation along the Pitch angle. However, after falling and flipping, the robot remains mobile, as can be observed in Video S7. The BFFSPR's slope climbing capability is also demonstrated in Video S8, Supporting Information, where the robot climbs a slope with a 12° inclination at a rate of 5.4BL s^−1^ (Figure 5C, Video S8, Supporting Information). Additionally, the robot can move while carrying irregular loads, as shown in Figure 6D. The weight of the robot is 0.058 g, and the load is an oval stone weighing 0.466 g, eight times that of the robot. The robot moves smoothly at a relative speed of 1.1 BL s^−1^ (Video S9, Supporting Information). Furthermore, the BFFSPR has potential application scenarios in temperature detection. As seen in Figure 6E, a temperature patch is attached to the robot. The patch is black below 55 °C and red above 55 °C. When the robot is placed on the left side of a heating table at 70 °C at 0s, the patch turns red as the robot moves onto the heating table at 24.93 s. When the robot moves to the right side of the heating table at 28.16 s, the temperature patch turns black again (Video S10, Supporting Information).

## 3 Conclusion

In conclusion, this study has developed a novel double spiral soft robot using a piezoelectric actuator to enhance the mobility, agility, and survivability of small soft robots in complex environments. The BFFSPR achieved a maximum average relative speed of 42.8 BL s^−1^ under the first resonance frequency and 200 Vpp voltage excitation, which is more than twice that of previously reported piezoelectric soft robots with strong maneuverability. When two BFFSPRs are attached in parallel to form a quadrupedal robot, it can achieve fast turns with a maximum turning speed of 482° s^−1^, surpassing the turning speed of most soft and rigid robots with good agility. The BFFSPR's motion capability outperforms all other reported soft robots. Moreover, due to its unique structural design, the BFFSPR can move quickly even after flipping, making it more adaptable to complex environments. The robot also performed well in climbing and weight‐bearing tasks, and we demonstrated its potential application for temperature detection. This work introduces a new structural design for traditional bipedal soft robots, which efficiently translates the bending of soft actuators into the swiftness of motion and environmental adaptability of soft robots, expanding the range of soft robot applications.

## 4 Experimental Section

### Fabrication of Bending Actuator

The active layer uses a 12‐micron‐thick PVDF film (PolyK Technologies, LLC), and the passive layer uses a conductive copper tape (Bao Jiaxin company). The fabrication processes of a prototype robot are shown in Figure S5, Supporting Information. the 12‐micron‐thick PVDF film and 50‐micron‐thick copper tape was cut into a 31 mm × 10 mm rectangular shape and attach the 7‐micron diameter silver wire to the copper tape, which was then gently attached to the PVDF film as shown in Figure S5a, Supporting Information. It was then attached the end of the other wire to the appropriate position of the silver electrode using a 3 mm × 3 mm copper tape as shown in Figure S5b, Supporting Information. Roll the pasted active layer and passive layer into a specified double spiral shape, and the soft robot was made.

### Robot Stimulation and Motion Characterization

A voltage signal was generated by an arbitrary waveform generator (AFG1022, Tektronic) and amplified up to 200 V (peak to peak voltage) by a voltage amplifier (HA 1600, PINTECH) to drive the soft robots. The motions of studied soft robotics were captured by a high‐speed camera (model FASTCAM Mini AX 100, Photron) with a sampling frequency of 4000 frames per second.

### Temperature Detection

The temperature‐reversible color change patch (Suzhou Hanqun Electronic Material Co., Ltd.) was pasted between the front and back legs of the robot. The temperature patch is black at room temperature and changes to red when the temperature reaches 55 °C.

## Conflict of Interest

The authors declare no conflict of interest.

## Author Contributions

E.C. designed and fabricated the prototype robots and experimental setup, performed the experiments, analyzed the data, and wrote the paper. Y.Y. wrote the paper and analyzed the results. B.L. performed high‐speed video recordings. G. L. assisted the experiment and analyzed the results. W. M. supervised the research and revised the paper. R.Y. developed the concept, directed the research, and revised the paper.

## Supporting information

Supporting Information

Supporting Information

Supporting Information

Supporting Information

Supporting Information

Supporting Information

Supporting Information

Supporting Information

Supporting Information

Supporting Information

Supporting Information

Supporting Information

## Data Availability

The data that support the findings of this study are available from the corresponding author upon reasonable request.

## References

[1] Z. Wang , K. Li , Q. He , S. Cai , Adv. Mater. 2019, 31, 1806849. 10.1002/adma.201806849 30575156

[2] Y. C. Cheng , H. C. Lu , X. Lee , H. Zeng , A. Priimagi , Adv. Mater. 2020, 32, 1906233. 10.1002/adma.201906233 31834665

[3] Z. Li , N. V. Myung , Y. Yin , Sci Robot 2021, 6, eabi4523. 34851711 10.1126/scirobotics.abi4523

[4] C. Li , G. C. Lau , H. Yuan , A. Aggarwal , V. L. Dominguez , S. Liu , H. Sai , L. C. Palmer , N. A. Sather , T. J. Pearson , D. E. Freedman , P. K. Amiri , M. O. de la Cruz , S. I. Stupp , Sci. Robot. 2020, 5, eabb9822. 33298516 10.1126/scirobotics.abb9822

[5] X. Peng , M. Urso , M. Ussia , M. Pumera , ACS Nano 2022, 16, 7615. 35451832 10.1021/acsnano.1c11136

[6] M. Z. Miskin , A. J. Cortese , K. Dorsey , E. P. Esposito , M. F. Reynolds , Q. Liu , M. Cao , D. A. Muller , P. L. McEuen , I. Cohen , Nature 2020, 584, 557. 32848225 10.1038/s41586-020-2626-9

[7] M. Zhu , W. Wang , C. Zhang , L. Zhu , S. Yang , Adv. Fiber Mater. 2021, 3, 172.

[8] C. Christianson , N. N. Goldberg , D. D. Deheyn , S. Cai , M. T. Tolley , Sci. Robot 2018, 3. 10.1126/scirobotics.aat1893 33141742

[9] W. B. Li , W. M. Zhang , Q. H. Gao , Q. Guo , S. Wu , H. X. Zou , Z. K. Peng , G. Meng , Soft Robot 2021, 8, 611. 33180656 10.1089/soro.2020.0012

[10] C. Tang , B. Du , S. Jiang , Q. Shao , X. Dong , X. J. Liu , H. Zhao , Sci Robot 2022, 7, eabm8597. 35613300 10.1126/scirobotics.abm8597

[11] X. Ji , X. Liu , V. Cacucciolo , Y. Civet , A. El Haitami , S. Cantin , Y. Perriard , H. Shea , Adv. Funct. Mater. 2020, 31, 2006639

[12] R. Chen , Z. Yuan , J. Guo , L. Bai , X. Zhu , F. Liu , H. Pu , L. Xin , Y. Peng , J. Luo , L. Wen , Y. Sun , Nat. Commun. 2021, 12, 7028. 34876570 10.1038/s41467-021-27265-w PMC8651723

[13] G. Li , X. Chen , F. Zhou , Y. Liang , Y. Xiao , X. Cao , Z. Zhang , M. Zhang , B. Wu , S. Yin , Y. Xu , H. Fan , Z. Chen , W. Song , W. Yang , B. Pan , J. Hou , W. Zou , S. He , X. Yang , G. Mao , Z. Jia , H. Zhou , T. Li , S. Qu , Z. Xu , Z. Huang , Y. Luo , T. Xie , J. Gu , et al., Nature 2021, 591, 66. 33658693 10.1038/s41586-020-03153-z

[14] Y. Liu , B. Chen , W. Li , L. Zu , W. Tang , Z. L. Wang , Adv. Funct. Mater. 2021, 31.

[15] G. Gu , J. Zou , R. Zhao , X. Zhao , X. Zhu , Sci. Robot 2018, 3, eaat2874. 33141690 10.1126/scirobotics.aat2874

[16] X. Ji , X. Liu , V. Cacucciolo , M. Imboden , Y. Civet , A. El Haitami , S. Cantin , Y. Perriard , H. Shea , Sci Robot 2019, 4, eaaz6451. 33137720 10.1126/scirobotics.aaz6451

[17] Y. Du , B. Peng , W. Zhou , Y. Wu , In 2022 IEEE 35th International Conference on Micro Electro Mechanical Systems Conference (MEMS) Tokyo, Japan , 2022, pp 644–647.

[18] Y. Wu , J. K. Yim , J. Liang , Z. Shao , M. Qi , J. Zhong , Z. Luo , X. Yan , M. Zhang , X. Wang , R. S. Fearing , R. J. Full , L. Lin , Sci Robot 2019, 4, eaax1594. 33137774 10.1126/scirobotics.aax1594

[19] J. Liang , Y. Wu , J. K. Yim , H. Chen , Z. Miao , H. Liu , Y. Liu , Y. Liu , D. Wang , W. Qiu , Z. Shao , M. Zhang , X. Wang , J. Zhong , L. Lin , Sci Robot 2021, 6, eabe7906. 34193563 10.1126/scirobotics.abe7906

[20] J. M. Liang , Y. C. Wu , Z. C. Shao , J. K. Yim , R. X. Xu , Y. Song , M. L. Qi , J. W. Zhong , M. Zhang , X. H. Wang , L. W. Lin , IEEE 32nd Int. Conf. Mechanical Systems (MEMS), Seoul, South Korea, January 2019, 1041.

[21] T. Park , Y. Cha , Sci. Rep. 2019, 9, 14700. 31605017 10.1038/s41598-019-51308-4 PMC6788992

[22] H. Lim , S.‐W. Kim , J.‐B. Song , Y. Cha , IEEE Access 2021, 9, 145477.

[23] C. Jin , J. Zhang , Z. Xu , I. Trase , S. Huang , L. Dong , Z. Liu , S. E. Usherwood , J. X. J. Zhang , Z. Chen , Adv. Intell. Syst. 2020, 2, 1900162.

[24] L. Qin , X. Liang , H. Huang , C. K. Chui , R. C. Yeow , J. Zhu , Soft Robot. 2019, 6, 455. 30883283 10.1089/soro.2018.0124

[25] J. Zhao , J. Zhang , D. McCoul , Z. Hao , S. Wang , X. Wang , B. Huang , L. Sun , Soft Robot. 2019, 6, 713. 31553262 10.1089/soro.2018.0098

[26] M. A. Aouraghe , Z. Mengjie , Y. Qiu , X. Fujun , Adv. Fiber Mater. 2021, 3, 38.

[27] Y. Zhao , Y. Chi , Y. Hong , Y. Li , S. Yang , J. Yin , Proc. Nat. Acad. Sci. U. S. A. 2022, 119, e2200265119. 10.1073/pnas.2200265119 PMC9295757 35605115

[28] Y. Y. Xiao , Z. C. Jiang , X. Tong , Y. Zhao , Adv. Mater. 2019, 31, 1903452. 10.1002/adma.201903452 31298439

[29] Y. Wang , Q. Guan , D. Lei , R. Esmaeely Neisiany , Y. Guo , S. Gu , Z. You , ACS Nano 2022, 16, 19393. 36367434 10.1021/acsnano.2c09066

[30] K. Liu , F. Hacker , C. Daraio , Sci Robot 2021, 6, eabf5116. 34043570 10.1126/scirobotics.abf5116

[31] S. Li , H. Bai , Z. Liu , X. Zhang , C. Huang , L. W. Wiesner , M. Silberstein , R. F. Shepherd , Sci Adv. 2021, 7, eabg3677. 34301600 10.1126/sciadv.abg3677 PMC8302124

[32] A. Kotikian , C. McMahan , E. C. Davidson , J. M. Muhammad , R. D. Weeks , C. Daraio , J. A. Lewis , Sci Robot 2019, 4, eaax7044. 33137783 10.1126/scirobotics.aax7044

[33] Y. Zhao , Y. Hong , F. Qi , Y. Chi , H. Su , J. Yin , Adv. Mater. 2022, 2207372. 10.1002/adma.202207372 36366927

[34] H. M. Peng , T. Mao , X. L. Lu , J. Intell. Mater. Syst. Struct. 2020, 31, 704.

[35] H. T. Lin , G. G. Leisk , B. Trimmer , Bioinspir. Biomim. 2011, 6, 026007. 21521905 10.1088/1748-3182/6/2/026007

[36] L. Wang , F. Zhang , Y. Liu , J. Leng , Adv. Fiber Mater. 2021, 4, 5.

[37] Q. Ze , S. Wu , J. Nishikawa , J. Dai , Y. Sun , S. Leanza , C. Zemelka , L. S. Novelino , G. H. Paulino , R. R. Zhao , Sci. Adv. 2022, 8, eabm7834. 35353556 10.1126/sciadv.abm7834 PMC8967224

[38] Q. Ze , S. Wu , J. Dai , S. Leanza , G. Ikeda , P. C. Yang , G. Iaccarino , R. R. Zhao , Nat. Commun. 2022, 13, 3118. 35701405 10.1038/s41467-022-30802-w PMC9198078

[39] Y. Kim , G. A. Parada , S. Liu , X. Zhao , Sci Robot 2019, 4, eaax7329. 33137788 10.1126/scirobotics.aax7329

[40] X. Fan , Y. Jiang , M. Li , Y. Zhang , C. Tian , L. Mao , H. Xie , L. Sun , Z. Yang , M. Sitti , Sci. Adv. 2022, 8, eabq1677. 36112686 10.1126/sciadv.abq1677 PMC9481141

[41] Y. Wu , X. Dong , J. K. Kim , C. Wang , M. Sitti , Sci. Adv. 2022, 8, eabn3431. 35622917 10.1126/sciadv.abn3431 PMC9140972

[42] Y. Kim , H. Yuk , R. Zhao , S. A. Chester , X. Zhao , Nature 2018, 558, 274. 29899476 10.1038/s41586-018-0185-0

[43] T. Xu , J. Zhang , M. Salehizadeh , O. Onaizah , E. Diller , Sci Robot 2019, 4, eaav4494. 33137716 10.1126/scirobotics.aav4494

[44] T. Wang , H. Ugurlu , Y. Yan , M. Li , M. Li , A. M. Wild , E. Yildiz , M. Schneider , D. Sheehan , W. Hu , M. Sitti , Nat. Commun. 2022, 13, 4465. 35915075 10.1038/s41467-022-32059-9 PMC9343456

[45] J. Zhang , R. H. Soon , Z. Wei , W. Hu , M. Sitti , Adv. Sci. 2022, 9, 2203730. 10.1002/advs.202203730 PMC9631051 36065052

[46] X. Yu , Z. Xie , Y. Yu , J. Lee , A. Vazquez‐Guardado , H. Luan , J. Ruban , X. Ning , A. Akhtar , D. Li , B. Ji , Y. Liu , R. Sun , J. Cao , Q. Huo , Y. Zhong , C. Lee , S. Kim , P. Gutruf , C. Zhang , Y. Xue , Q. Guo , A. Chempakasseril , P. Tian , W. Lu , J. Jeong , Y. Yu , J. Cornman , C. Tan , B. Kim , et al., Nature 2019, 575, 473. 31748722 10.1038/s41586-019-1687-0

[47] R. Yin , B. Yang , X. Ding , S. Liu , W. Zeng , J. Li , S. Yang , X. Tao , Adv. Mater. Technol. 2020, 5, 2000341.

[48] Y. Chi , Y. Hong , Y. Zhao , Y. Li , J. Yin , Sci. Adv. 2022, 8, eadd3788. 36399554 10.1126/sciadv.add3788 PMC9674291

[49] J. Fan , S. Wang , Q. Yu , Y. Zhu , Soft Robot 2020, 7, 615. 32401696 10.1089/soro.2019.0094

[50] T. J. Jones , E. Jambon‐Puillet , J. Marthelot , P. T. Brun , Nature 2021, 599, 229. 34759362 10.1038/s41586-021-04029-6

[51] S. Zhuo , Z. Zhao , Z. Xie , Y. Hao , Y. Xu , T. Zhao , H. Li , E. M. Knubben , L. Wen , L. Jiang , M. Liu , Sci. Adv. 2020, 6, eaax1464. 32064332 10.1126/sciadv.aax1464 PMC6994219

[52] W.‐B. Li , X.‐Y. Guo , W.‐M. Zhang , IEEE Robot Autom Lett 2021, 6, 1654.

[53] G. Wang , M. Zhu , Adv. Fiber Mater. 2021, 3, 381.

[54] M. M. Coad , L. H. Blumenschein , S. Cutler , J. A. R. Zepeda , N. D. Naclerio , H. El‐Hussieny , U. Mehmood , J.‐H. Ryu , E. W. Hawkes , A. M. Okamura , V. Robots , IEEE Rob. Autom. Mag. 2020, 27, 120.

[55] R. J. Bomphrey , T. Nakata , N. Phillips , S. M. Walker , Nature 2017, 544, 92. 28355184 10.1038/nature21727 PMC5412966

[56] H. Fang , K. W. Wang , J. Sound Vib. 2017, 391, 153.

[57] A. Erturk , D. J. Inman , J. Vib. Acoust 2008, 130, 041002.

[58] J. K. Schonebaum , F. Alijani , G. Radaelli , Proc. Inst. Mech. Eng., Part C 2021, 235, 7907.

[59] N. T. Jafferis , E. F. Helbling , M. Karpelson , R. J. Wood , Nature 2019, 570, 491. 31243384 10.1038/s41586-019-1322-0

[60] H. Peng , J. Yang , X. Lu , P. Zhu , D. Wu , IEEE Trans Ind Electron 2019, 66, 7852.

[61] Y. Chen , H. Zhao , J. Mao , P. Chirarattananon , E. F. Helbling , N.‐s. P. Hyun , D. R. Clarke , R. J. Wood , Nature 2019, 575, 324. 31686057 10.1038/s41586-019-1737-7

[62] D. Wang , Y. Liu , J. Deng , S. Zhang , J. Li , W. Wang , J. Liu , W. Chen , Q. Quan , G. Liu , H. Xie , J. Zhao , Adv. Sci. 2022, 9, 2203054. 10.1002/advs.202203054 PMC9561757 35981889

[63] T. Kamimura , S. Aoi , Y. Higurashi , N. Wada , K. Tsuchiya , F. Matsuno , Sci. Rep. 2021, 11, 9631. 33953253 10.1038/s41598-021-88879-0 PMC8099890

[64] A. M. Wilson , T. Y. Hubel , S. D. Wilshin , J. C. Lowe , M. Lorenc , O. P. Dewhirst , H. L. A. Bartlam‐Brooks , R. Diack , E. Bennitt , K. A. Golabek , R. C. Woledge , J. W. McNutt , N. A. Curtin , T. G. West , Nature 2018, 554, 183. 29364874 10.1038/nature25479

[65] J. E. Bertram , A. Gutmann , J. R. Soc. Interface 2009, 6, 549. 18854295 10.1098/rsif.2008.0328 PMC2696142

[66] Y. Zeng , S. Crews , J. Exp. Biol. 2018, 221, jeb166512. 29440135 10.1242/jeb.166512

[67] R. M. Walter , J. Exp. Biol. 2003, 206, 1739. 12682105 10.1242/jeb.00349

[68] S. Wohl , S. Schuster , J. Exp. Biol. 2007, 210, 311. 17210967 10.1242/jeb.02646

[69] S. Rubin , M. H. Young , J. C. Wright , D. L. Whitaker , A. N. Ahn , J. Exp. Biol. 2016, 219, 676. 26787481 10.1242/jeb.128652

[70] X. Zhang , J. Yan , K. Yang , J. Zhao , S. Tang , IEEE Robot Autom Lett. 2022, 7, 2463.

[71] B. Liu , F. L. Hammond , in 2020 IEEE/RSJ International Conference on Intelligent Robots and Systems (IROS), Las Vegas, NV, USA , 2020, pp.1705–1710.

