# Supplementary material for: Bio‐Mimic, Fast‐Moving, and Flippable Soft Piezoelectric Robots
Source: Adv Sci (Weinh). 2023 May 10;10(20):2300673. doi: 10.1002/advs.202300673 (PMC10369280; doi:10.1002/advs.202300673)
Supplement: Supplementary file 1 — Supporting Information [file ADVS-10-2300673-s001.pdf]

ADVANCED SCIENCE
Open Access

Supporting Information

for *Adv. Sci.*, DOI 10.1002/advs.202300673

Bio-Mimic, Fast-Moving, and Flippable Soft Piezoelectric Robots

Erdong Chen, Yiduo Yang, Mengjiao Li, Binghang Li, Guijie Liu, Weilei Mu and Rong Yin**

Supplementary Information

Bio-Mimic, Fast-Moving, and Flappable Soft Piezoelectric Robots

Authors: Erdong Chen[1,2], Yiduo Yang[2], Mengjiao Li[1,2], Binghang Li[1,2], Guijie Liu[1], Weilei Mu[1*], Rong Yin[2*]

Affiliations:

[1]College of Engineering, Ocean University of China, Qingdao, 266100, China
[2]Textile Engineering, Chemistry and Science, Wilson College of Textiles, North Carolina State University, Raleigh, NC 27695, USA
*Corresponding author. Email: muweilei@ouc.edu; ryin@ncsu.edu

Supplementary Text

Text S1. Selection of PVDF film thickness

To optimize geometric parameters, we have considered the maximum curvature as the object function. The curvature of the composite structure is [17]:

$$\frac{1}{R}=\frac{3[ct_3^2(E_3-E_2)-E_1t_1(c-\varepsilon)(2t_2+t_1)]}{E_3t_3^2(2t_3+3t_b)+E_2t_2(2t_2^2-3t_2t_b)+E_1t_1(6t_2^2+6t_1t_2-6t_2t_b+2t_1^2-3t_1t_b)}$$

Where U is the driving voltage, d_{31} is the piezoelectric constant; t_1, t_2 and t_3 are the thickness of PVDF film, adhesive layer and copper tape; E1, E2 and E3 are young's modulus of these layers; $\varepsilon=\frac{Ud_{31}}{t_1}$; $c=\frac{E_1t_1\varepsilon}{E_3t_3+E_2t_2+E_1t_1}$; $t_b=\frac{-E_3t_3^2+E_2t_2^2+E_1t_1(2t_2+t_1)}{2(E_3t_3+E_2t_2+E_1t_1)}$. With the given copper tape thickness and other material parameters listed in Table S1, the relationship between the PVDF layer thickness and curvature is shown in Fig. S1(d). It can be concluded that the smaller the thickness of PVDF, the larger the curvature. For verification, we utilized COMSOL Multiphysics to construct a 3D dynamic finite element model of the piezoelectric actuator, which is illustrated in Figure S1(a). The top of the PVDF film was prescribed a voltage, while the bottom of the PVDF film was grounded. The polarization direction of PVDF is set to the Z-axis direction. The left sides of the actuator were fixed as a mechanical boundary condition. The model was run as a series of simulations. Simulations were run for actuators of several different geometries. The PVDF of thickness of 12mm, 20mm, 30mm, and 40mm was tested. PVDF is electrically excited with a frequency of 1-40Hz and a peak of 200Vpp,

simulation run and used to generate the plots in Figure S1(d). It can be concluded that as the thickness of PVDF decreases, the amplitude increases, which is consistent with the trend of the curvature calculation above. As a compromise, a 12-um-thick PVDF film was chosen from among the vendor's products.

Table S1. Resonance frequency of each order.

	PVDF	Adhesive	Copper
thickness	t_1=12μm	t_2=30μm	t_3=20μm
width	w_1=10mm	w_2=10mm	w_3=10mm
Young's modulus	E_1=2.8~3.2GPa	E_2=1~50MPa	E_3=90~123.5GPa
density	ρ_{123}=1.78g/cm^3	ρ_{123}=1.2g/cm^3	ρ_{123}=8.96g/cm^3
piezo strain constant	d_{31}=30Pc/N		
piezo stress constant	Gg_{31}=0.21Vm/N		
relative permittivity	ε_{r1}=13	ε_{r2}=3.3	ε_{r3}=13
poison ratio	ν_1=0.34~0.39	ν_2=0.47~0.49	ν_3=0.32~0.42

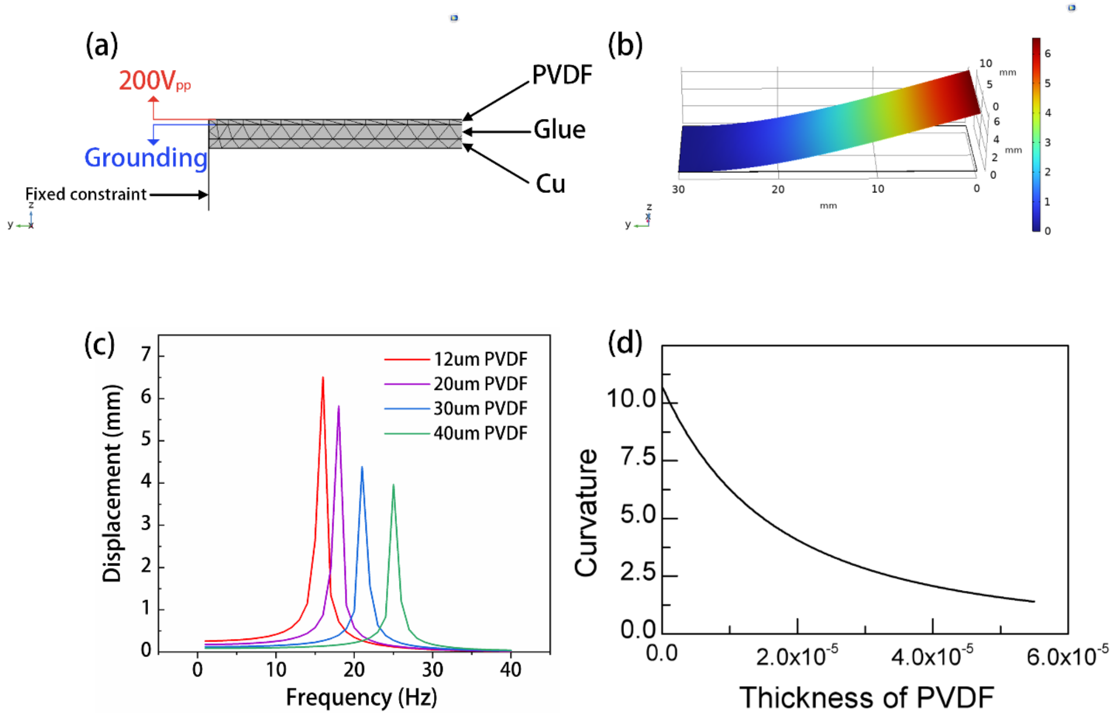

Figure S1 Selection of PVDF film thickness. (a) Mesh and boundary condition settings for finite element models. (b) Bending deformation from finite element calculations of piezoelectric actuators. (c) Relationship between the excitation frequency and amplitude of piezoelectric actuators composed of several different thicknesses of PVDF film at 200Vpp. (d)Variation of curvature with PVDF thickness at three-layer thicknesses.

Text S2.FEM Simulation detail

We imported the 3D geometry model into COMSOL Multiphysics. The material settings are similar to the previous section, and the mechanical boundary conditions are set to free. In the modal simulation, we study the resonant frequencies of the soft robot. The calculated first 10 orders of modes are shown in Table S2 and the modal motions in the main operating frequency range of the robot are investigated, as well as the displacement of the four main points a,b,c,d that interact with the ground as a function of the excitation frequency shown in Figure S2.

Table S2. Resonance frequency of each order.

Order Number	Resonance frequency
1	176.5
2	196.6
3	222.01
4	304.72
5	374.04
6	463.46
7	491.89
8	656.84
9	851.67
10	1073.8

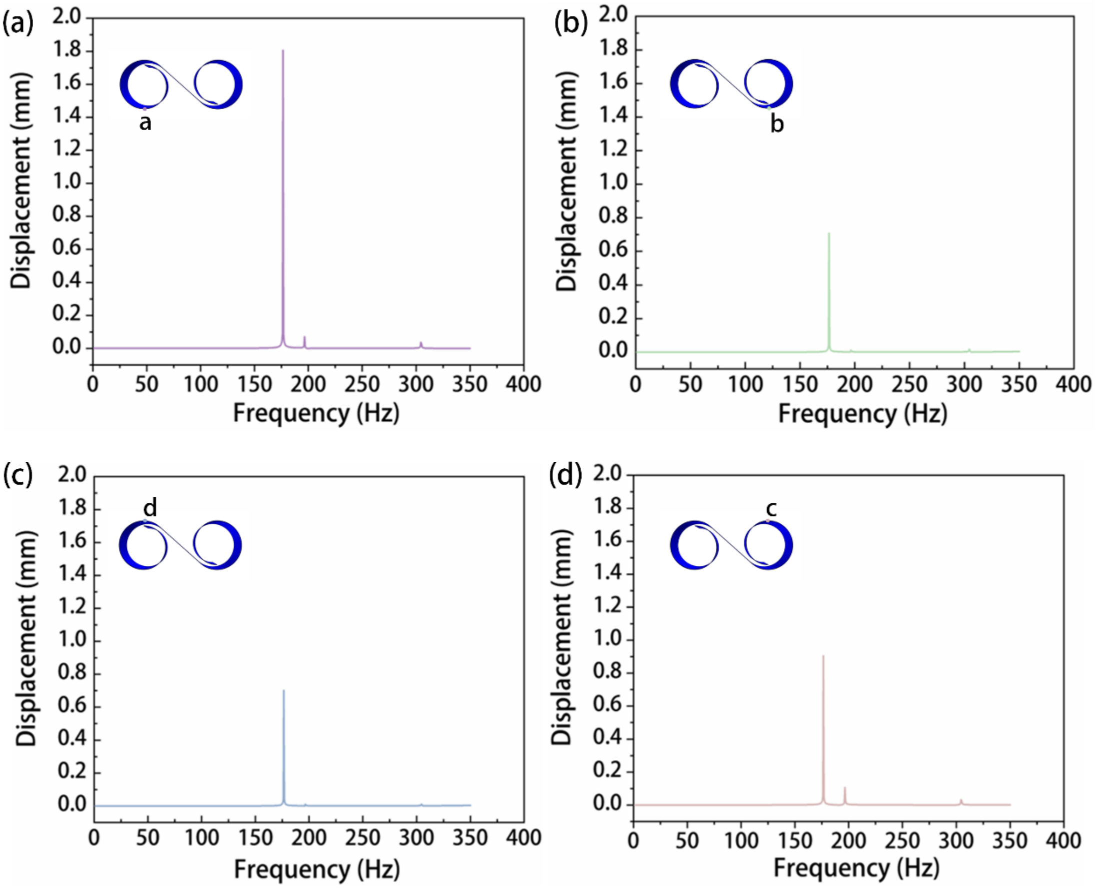

Figure S2. The relationship between the displacement of the four working points and the excitation frequency a-d.

Text S3. Backward motion

We found that the soft-bodied robot exhibited the opposite motion speed of the robot under electrical excitation at 300 Hz. It is speculated that the motion of the robot is closely related to its modal at each resonance frequency. The finite element simulation results show the deformation of the robot using a voltage excitation near the fourth order resonant frequency (304.5 Hz) of the robot as shown in Figure S3(a). It is clear that the deformation of the robot is different from that at the first intrinsic frequency(176.5 Hz). The deformation of the robot is mainly focused on the contraction and expansion in the body length direction, but the amplitude of this mode is much smaller than that of the first intrinsic frequency mode. The same phenomenon can be observed by observing the video of the robot taken under the high-speed camera, where the reduction in amplitude and the change in mode result in a lower speed and a backward direction of motion of the robot. The force analysis of the backward motion of the robot is shown in Figure S3(b).

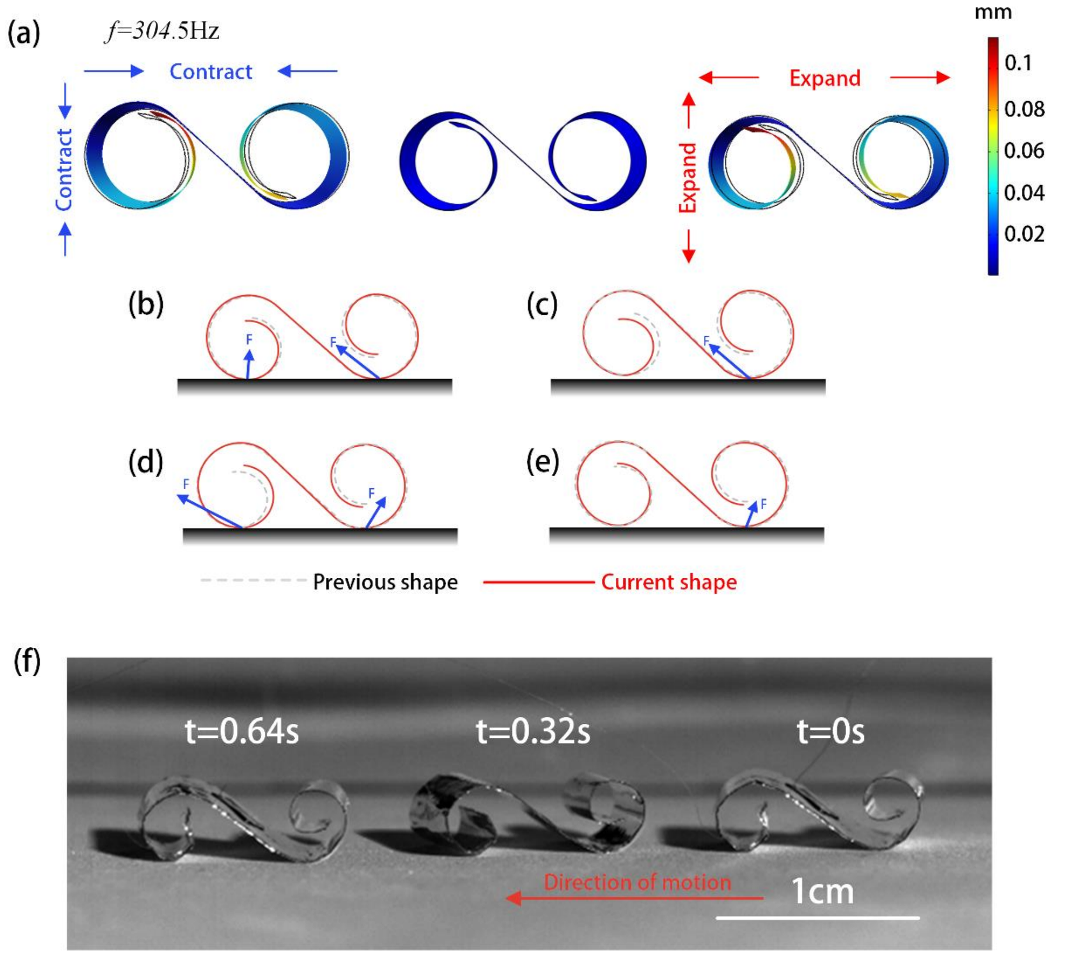

Figure S3. Mechanism of robot backward motion. (a) The FEM result of the vibration mode at the fourth resonant frequency (b)Show the gait and force analysis with cross-sectional views of the BFFSPR's corresponding contraction (top) and expansion (bottom) in four different states (contraction at forefoot touchdown, expansion at forefoot touchdown, contraction at both foot touchdown and expansion at both foot touchdown).(f) Real motion process according to frame-by-frame high-speed video in 0.64 s with 200Vpp and fourth resonant frequency Electrical excitation.

Text S4. Simplified dynamic model for the robot's locomotion

Parameters (**Fig. S4**):

Mass: m_1, I_1,m_2, I_2

Kinematics: l_{1x}, l_{1y}, l_{2x}, l_{2y}, l_{12}, l_{21},θ_1, θ_2, θ_r

Forces: d, k, d_x, d_θ, k_θ, τ_m

Control parameters: f

According to the high-speed video and the modal simulation summary of FEM, the expansion and contraction of the first-order modes of the soft robot are the main vibration modes, and there is almost no deformation of the robot in the width direction, and the higher-order vibration modes can be ignored. Therefore, the next simulation only considers the first mode deformation of the robot in the x-z plane.

Therefore, we propose a planar mass-spring model which is shown in Fig.S4. The expansion and contraction of the robot is considered as the extension and bending of

the central hinge. The soft-body robot is considered as the extension and bending of a central hinge, and the rigid body represents the front and rear halves of the robot with front and rear masses m_1 and m_2, respectively, and rotational inertia I_1 and I_2. The free-body diagrams based on two possible poses (air and back legs touching the ground) with applied ground reaction forces are shown in Fig.S4

The configuration of the system is described by the following coordinates: the coordinates x, y of the front center of gravity (G), and the angles θ_1 and θ_2 of the front and back bodies relative to the horizontal. the state vector X is the 8-dimensional vector[x ,y ,θ_1 ,θ_2,] The interaction of the soft robot feet with the ground is modeled as a vertically acting spring damper (k-d) and a horizontally acting frictional force. The normal force is computed by

$$F_{nF} = sign(-y_{FN})sign(-ky_{FN} - d\dot{y}_{FN})(-ky_{FN} - d\dot{y}_{FN})$$

Where (x_{FN}, y_{FN}) is the position of the foot, N=1 is the front leg and N=2 is the rear leg of the robot. When the foot is below the ground and the vertical force is positive, this normal force acts as a spring dampener (k-d). Otherwise the force is zero. These foot coordinates and the velocities of the foot points are succinctly omitted) can be calculated from the state vectors by the robot's kinematics as follows.

$$x_{F1}=x+l_3sin\theta_1$$

$$y_{F1}=y-l_3cos\theta_1$$

$$x_2=x-(l_1cos\theta_1+l_2cos\theta_2)$$

$$y_2=y-(l_1sin\theta_1-l_2sin\theta_2)$$

$$x_{F2}=x_2-l_4sin\theta_2$$

$$y_{F2}=y_2-l_4cos\theta_2$$

The horizontal Coulomb friction is：

$$F_{fN}(X) = sign(-\dot{x}_{FN})\min(|d_x \dot{x}_{FN}|,\mu,{}_{nN})$$

where μ is the coefficient of friction between the foot and the ground and dx is the slope of the friction. The torque at the joint is: Torque assumes that the motor torque is sinusoidal:

$$\tau(t) = \tau_m \sin 2\pi ft - k_\theta(\theta_2 - \theta_1 - \theta_r) - d_\theta(\dot{\theta}_2 - \dot{\theta}_1)$$

where τm is the amplitude of the motor torque, which varies sinusoidally with time, and θr is the angle of repose of the curve. The Newtonian equations for the robot acceleration and angular acceleration are as follows:

$$
\begin{bmatrix}
m_1 & 0 & 0 & 0 & 1 & 0 \\
0 & m_1 & 0 & 0 & 0 & 1 \\
0 & 0 & -I_1 & 0 & l_1 \sin\theta_1 & -l_1 \cos\theta_1 \\
m_2 & 0 & l_1 m_2 \sin\theta_1 & l_2 m_2 \sin\theta_2 & 1 & 0 \\
0 & m_2 & -l_1 m_2 \cos\theta_1 & l_2 m_2 \cos\theta_2 & 0 & 1 \\
0 & 0 & 0 & I_2 & l_2 \sin\theta_2 & l_2 \cos\theta_2
\end{bmatrix}
\begin{bmatrix}
\ddot{x} \\ \ddot{y} \\ \ddot{\theta}_1 \\ \ddot{\theta}_2 \\ F_{px} \\ F_{py}
\end{bmatrix}
=
$$

$$
\begin{bmatrix}
F_{f1}(X) \\
m_1 g - F_{n1}(X) \\
-\tau(t) + F_{f1}(X)(l_3 \cos\theta_1) - F_{n1}(X)(l_3 \sin\theta_1) \\
F_{f2}(X) - m_2(\omega_1^2 l_1 \cos\theta_1 + \omega_2^2 l_2 \cos\theta_2) \\
m_2 g - F_{n2}(X) - m_2(\omega_1^2 l_1 \sin\theta_1 + \omega_2^2 l_2 \sin\theta_2) \\
-\tau(t) + F_{n2}(X)(l_4 \sin\theta_2) - F_{f2}(X)(l_4 \sin\theta_2)
\end{bmatrix}
$$

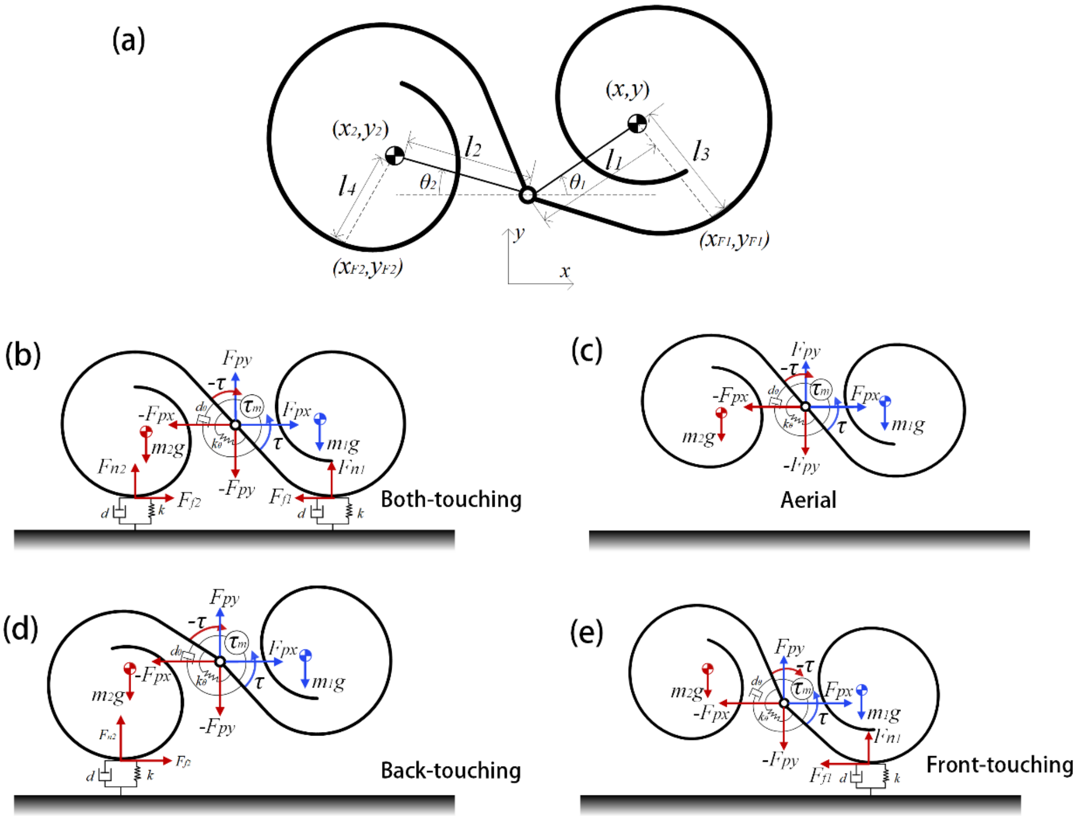

Figure S4. System configurations of the simplified dynamic model. (a) Dimensions of the simplified dynamic model. (b)-(e) Free body diagram of the simplified dynamic model in the postures of aerial, front-touching, back-touching and both-touching, respectively.

Table S3. Comparison of maximum running and turning speeds of animals, soft and rigid robots.

Category	Relative speed (BL/s)	Turning speed(°/s)	Reference	Flippable(Y/N)
Mite	192.4	795	(65)	No
Flattie spiders	20.833	2500	(62)	No
Cheetah	26.4	76	(60)	No
Mouse	35.7	1350	(63)	No
Impala	18.3	66.83	(60)	No
Zebra	7	31.5	(60)	No
Xiphias gladius	20	4500	(64)	No
Lion	8.8	40.86	(60)	No
Motor	2	15	(46)	No
DEA	6.01	150	(11)	No
Electrostatic	0.533	45	(14)	No
Electrostatic	1.04	62.79	(22)	No
DEA	0.3	30	(15)	No
Motor	0.13	15.09	(23)	No
PVDF	2.33	102.6	(19)	No
PVDF	7.5	650	(18)	No
motor	1.4	45	(66)	No
motor	0.08	23.6	(50)	No
motor	0.37	41.6	(67)	No
motor	10.9	524.4	(58)	No
SMA	3.5	25.7	(32)	No
PVDF	42.8	482	This work	Yes

Text S5. Fabrication processes of a prototype robot

We cut the PVDF film with silver plated electrodes on two sides into a PVDF strip of 30mm×10mm. The copper tape with acrylic adhesive was cut into the strip with the same size as the PVDF strip. We sticked the silver wire to the acrylic adhesive layer of the copper tape, and attached the PVDF strip to the copper strip to obtain a composite strip. After setting, another silver wire is glued on the other side of PVDF. The body length of the soft robot is 10mm, R is 2mm, and B is 10mm, as shown in Figure S5. Similarly, two composite strips can be made into a dual-body soft robot by joining them with PI tape, as shown in Figure S6.

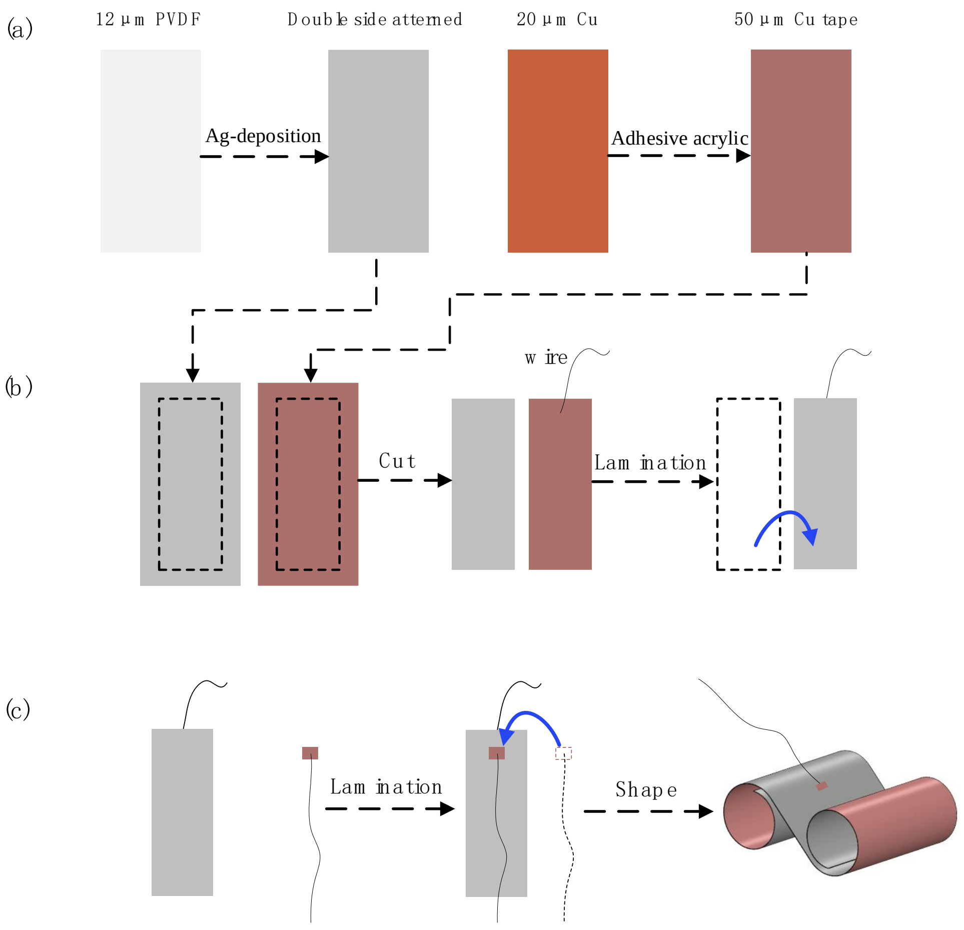

Figure S5. The process of making a soft robot.

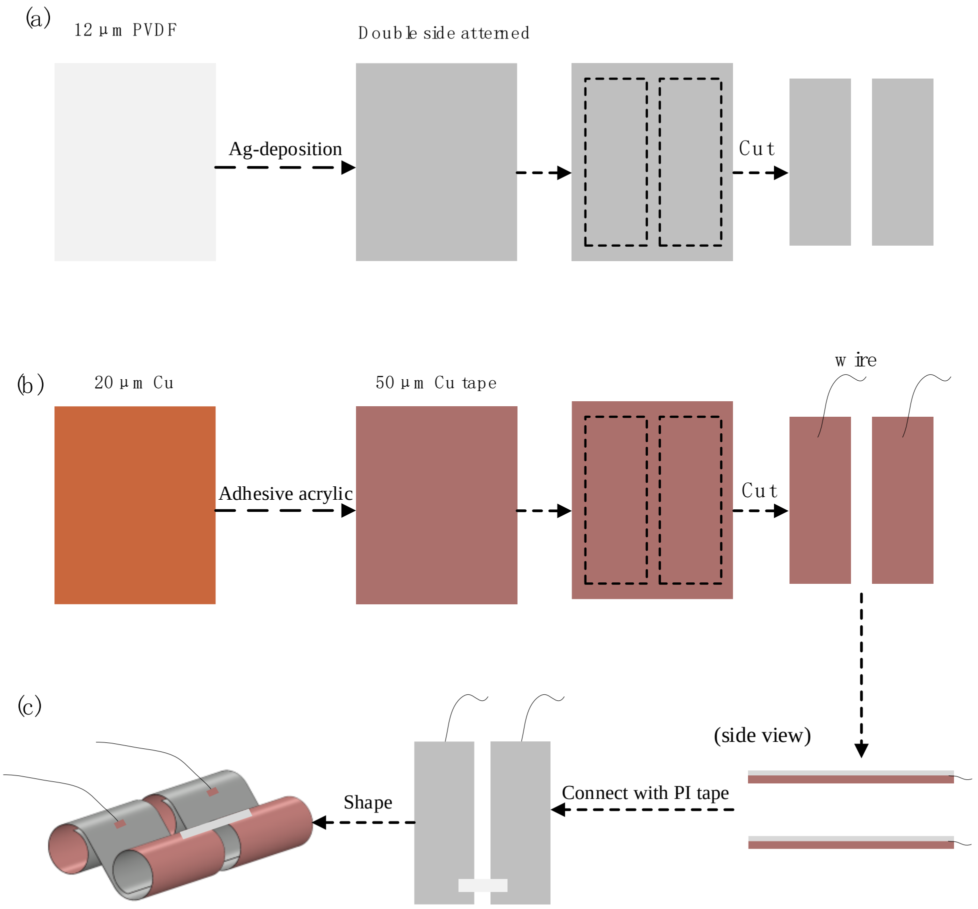

Figure S6 The process of making a dual-body soft robot.

Text S6. The qualitative analysis of the frictional force

The qualitative analysis of the frictional force generated in the two gaits of Back-touching in expansion and Front-touching in contraction during the movement of BFFSPR is shown in FigS7.A and FigS7.B, respectively. In FigS7.A, point B represents the point where the hind leg contacts the ground, while in FigS7.B, point A represents the point where the front leg contacts the ground, and COG represents the center of gravity of the robot. The horizontal and vertical speed of the *COG* is defined as V_h and V_g, respectively. The vibration of the soft robot near the resonance results in an expanding or contracting speed V_f at point A and B, and its magnitude and direction are a function of time. It is noted that average V_f has a much larger magnitude than V_g and V_h under the resonant frequency. This is consistent with the observation from the high-speed video (Video S1): the robot undergoes several resonant cycles between ground impacts. Therefore, the absolute velocity of the contact points between the soft robot and the ground is the sum of V_h, V_g, and V_f(shown in the orange-shaded area in FigS7.A and FigS7.B). The reaction force between the robot and ground is hard to measure but should be opposite to the absolute velocity at point A and B under the assumption of rigid impact of the foot-ground interaction. And the magnitude of F_f is approximately proportional to the absolute velocity at point A and B, leading to a blue-

shaded area in FigS7.A and FigS7.B.

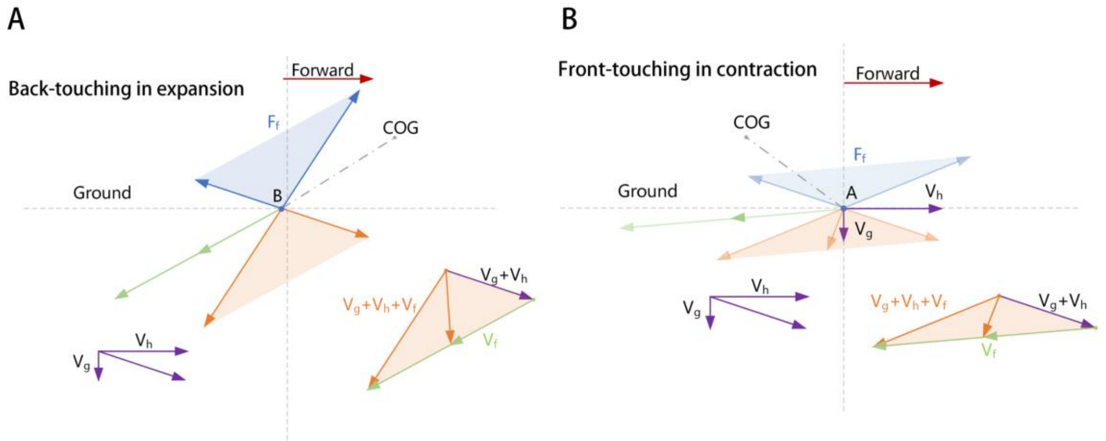

Figure S7 Frictional force analysis of the two motion gaits.

Text S7. Motion experiment under wind load

The wind load is generated by a fan, and the direction of the wind is aligned with the direction of motion of the BFFSPR. An anemometer (RA310) is placed behind the BFFSPR to measure the wind speed, as shown in Fig. S8.

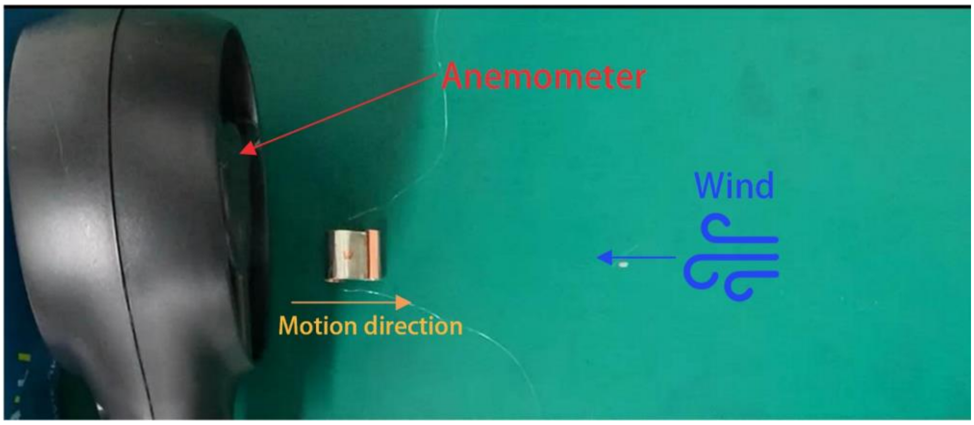

Figure S8 The schematic diagram of the BFFSPR's motion experiment under wind load.

Text S8. Robustness

When a brick 20 times the weight of the BFFSPR falls on the robot from a distance of 5cm above the desktop, the robot can move forward at a speed of 3BL/s after being impacted, as shown in the FigS9. The reason for the decrease in the motion speed of BFFSPR is that, after being subjected to impact, its back legs change from a spiral shape to a flat oval shape. Although the BFFSPR will undergo plastic deformation and reduce its motion performance, its original shape and motion performance can be restored by simple shaping.

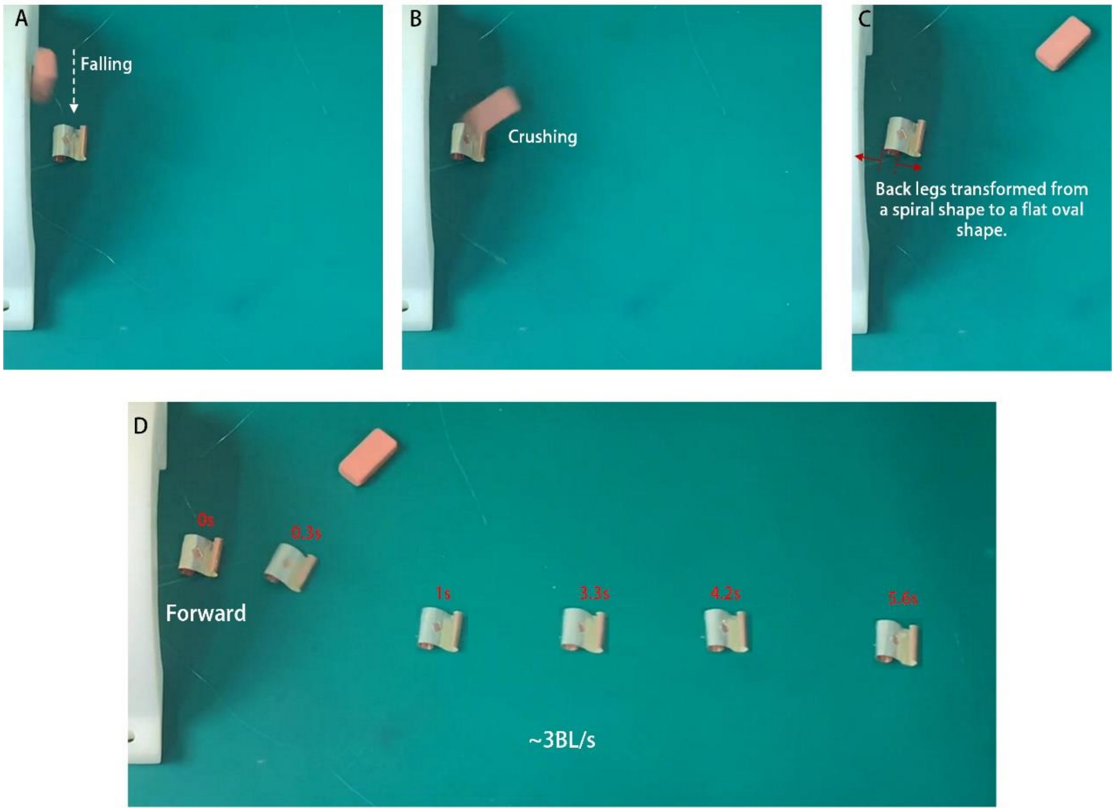

Figure S9 Robustness demonstration.
